# Minimal Change Disease: A Case of Delayed Recognition Due to Chronic Lower Extremity Edema

**DOI:** 10.7759/cureus.105667

**Published:** 2026-03-22

**Authors:** Grant Corven, Sarah Baker, Delmis Perez Castro, Andrew Kossack

**Affiliations:** 1 Internal Medicine, Naples Comprehensive Health (NCH) Healthcare System, Naples, USA

**Keywords:** edema, lymphedema, minimal change disease (mcd), nephrology, nephrotic syndrome, podocyte effacement, proteinuria

## Abstract

We present the case of a 79-year-old woman with new-onset nephrotic syndrome discovered by working up worsening lower extremity edema. Renal biopsy showed a pathology report consistent with minimal change disease (MCD). The patient was treated with prednisone and, subsequently, tacrolimus with good clinical response and improvement in her serum albumin and total protein levels upon outpatient follow-up. This case underscores the importance of a broad differential for the common complaint of lower extremity edema and the additional risks of MCD and adverse effects of its treatment.

## Introduction

Nephrotic syndrome is characterized by excessive proteinuria (>3.5 g in 24 hours), generalized edema, low albumin, and hyperlipidemia [1]. Minimal change disease (MCD) accounts for 70-90% of pediatric nephrotic syndrome [2] but only 10-15% of primary nephrotic syndrome in adults, behind focal segmental glomerular sclerosis and membranous nephropathy [3]. Clinical presentation of MCD is consistent with typical nephrotic syndrome, including edema (most commonly of the lower extremities, scrotum/labia, periorbital) or possibly anasarca, which can include pleural or pericardial effusions [4]. The exact pathophysiology is generally unknown, but treatment is centered around steroids or additional immunosuppression if refractory to steroids. Forms that are sensitive to steroids generally have more frequent relapses but a lower risk of advanced renal dysfunction [4]. Early identification of the cause of nephrotic syndrome in adults is crucial in developing a therapeutic approach. We present a case of new-onset MCD in a 79-year-old woman with chronic lower extremity edema.

## Case presentation

A 79-year-old woman with a past medical history of paroxysmal atrial fibrillation on apixaban, chronic venous insufficiency, and hypertension was admitted to the general medicine floor of Naples Comprehensive Health (NCH) Baker Hospital with bilateral lower extremity edema that had been worsening for the last three weeks. The patient had a history of chronic lower extremity edema and had been diagnosed with chronic venous insufficiency, but stated that the swelling was increasing and she was gaining weight. She was recently seen by her primary care physician (PCP), who prescribed oral furosemide 20 mg twice daily for three days; however, this did not improve her symptoms. Eventually, she developed weeping fluid from her legs and anasarca up to the abdomen with associated shortness of breath, leading to her presentation in the hospital. She also noted “foamy urine” before presentation. Upon admission, her vital signs were a blood pressure of 158/77 mmHg, a pulse rate of 75 beats/minute, a temperature of 35.8°C, a respiratory rate of 16 breaths/minute, and an oxygen saturation (SpO_2_) of 97% on room air. Physical examination revealed no acute distress and no cardiac murmurs or rhythm abnormalities. Crackles were auscultated in the left lower lung field. Abdominal examination revealed anasarca to the level of the umbilicus with pitting edema from the abdomen to the feet bilaterally. Cutaneous examination was remarkable for a faint papular rash on the anterior shins bilaterally with evidence of venous stasis changes.

Laboratory investigations revealed slight hemoconcentration with a hemoglobin of 16.4 g/dL and a hematocrit of 50.2%. There was no leukocytosis. Electrolytes were within normal limits. Additional results revealed a blood urea nitrogen of 28 mg/dL and creatinine of 0.8 mg/dL. Calcium was 8.7 mg/dL. Aspartate aminotransferase and alanine aminotransferase were within normal laboratory limits. Albumin was 0.9 g/dL, and total protein was 6.0 g/dL. Given the anasarca and rash on cutaneous examination, hepatitis titers were collected to rule out cryoglobulinemia. Hepatitis B surface and core antibodies were both positive, and hepatitis B surface and e antigens were both negative, indicating likely previous infection and recovery. Hepatitis C antibody screening was negative. The fact that her liver function tests, international normalized ratio, and platelets were all within normal limits led us to have low suspicion for a hepatic cause. Transthoracic echocardiography was performed to rule out a cardiogenic cause and showed a preserved ejection fraction with no wall motion abnormalities or evidence of heart failure.

Urine studies and serum protein electrophoresis were ordered, along with complement levels, to assess for nephrotic syndrome. Complement C3 was 202 mg/dL, and C4 was 31.2 mg/dL. Serum protein electrophoresis showed a total protein of 4.8 g/dL, albumin of 1.5 g/dL, alpha 1 globulin of 0.2 g/dL, alpha 2 globulin of 1.1 g/dL, and a faint band in the beta region was noted by the pathology lab. Urine protein electrophoresis showed a total protein of 462.2 mg/dL, with albumin of 67.5%. Reflex serum immunofixation showed a polyclonal gammopathy (IgA: 522 mg/dL, IgM: 238 mg/dL, IgG: 654 mg/dL) with no observed M-spike. Ant-GBM antibody testing was negative. Urine protein testing was 1571.7 mg/dL (the upper limit of normal in our reference laboratory is 12 mg/dL), and the urine protein/creatinine ratio was 6.8. With consultation by nephrology, the patient was continued on diuretic therapy for the edema with furosemide and spironolactone due to intermittent episodes of hypokalemia during the hospital stay. A renal biopsy was pursued to specify the cause of her nephrotic syndrome before initiating treatment. The patient was discharged from the hospital in stable condition on diuretic therapy while awaiting results of the tissue pathology.

**Figure 1:**
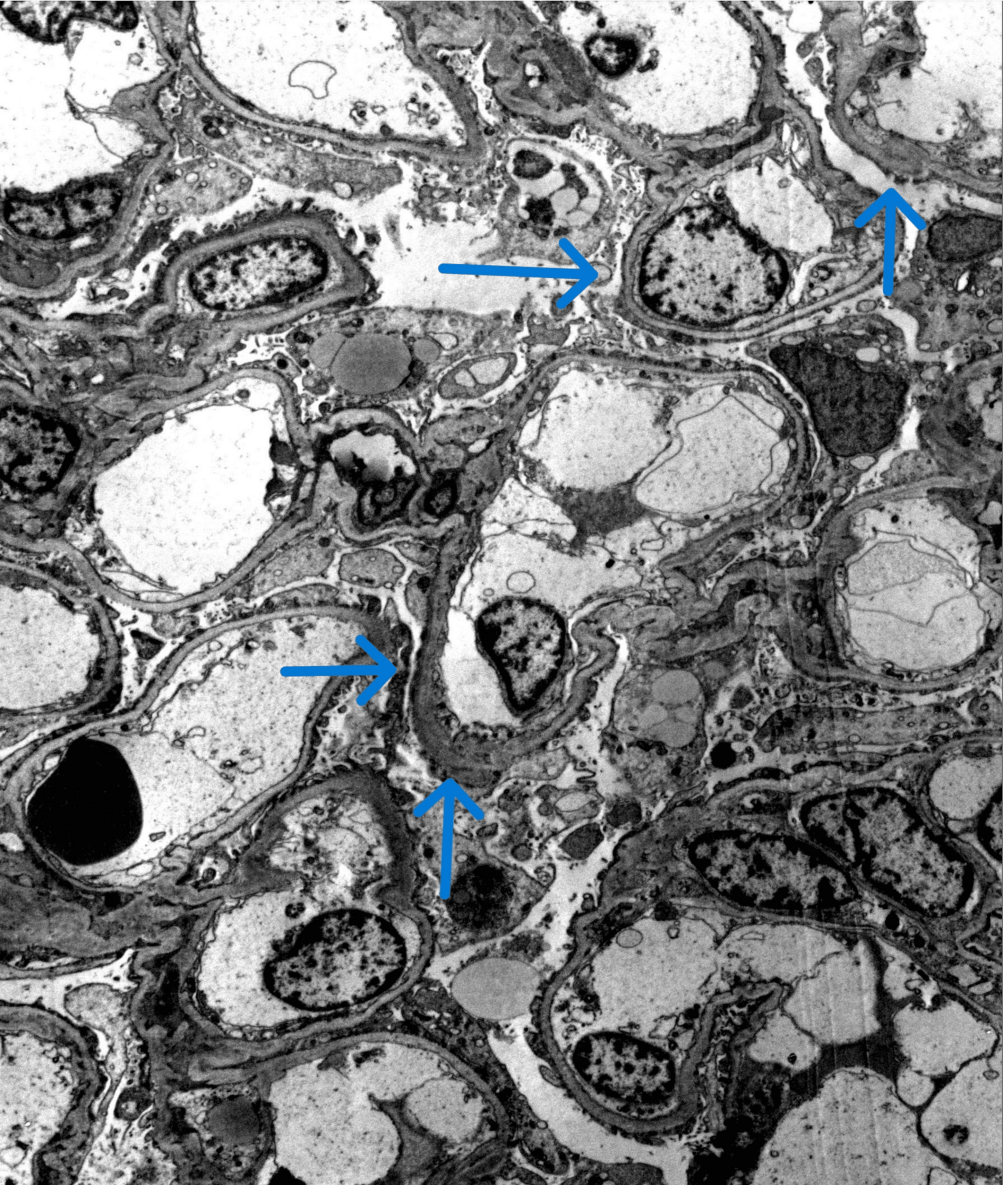
Transmission electron microscopy showing extensive podocyte foot process effacement. Image credit: Arkana Laboratories, 2024.

**Figure 2:**
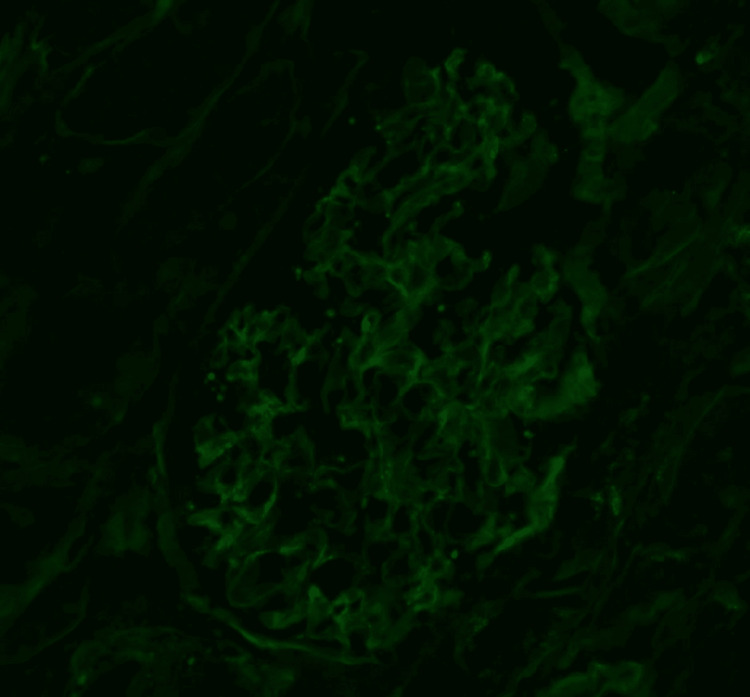
Negative immunofluorescent staining demonstrating a lack of immunoglobulin or complement deposition. Image credit: Arkana Laboratories, 2024.

Pathology report obtained after discharge through outpatient records showed severe epithelial foot process effacement with areas of microvillous transformation of the podocytes on electron microscopy (Figure 1). Additionally, immunofluorescence staining was negative for immunoglobulin or complement deposition, which distinguishes MCD from immune-mediated glomerular disease (Figure 2).

This confirmed a diagnosis of MCD as the cause of her nephrotic syndrome. This information was communicated to the patient’s PCP along with further recommendations from nephrology to begin the patient on 60 mg of oral prednisone daily, oral trimethoprim-sulfamethoxazole for *Pneumocystis jirovecii* pneumonia prophylaxis, and an oral proton pump inhibitor for gastrointestinal prophylaxis while on prednisone. The initial therapy followed current guidelines for the treatment of initially diagnosed MCD with prednisone therapy (1 mg/kg/day, max 80 mg daily). The initial plan was to treat until complete remission (up to 16 weeks) and then begin to taper the prednisone.

A review of outpatient records about eight weeks after discharge showed that the patient was beginning to taper the prednisone dose and had been started on tacrolimus by her outpatient nephrologist due to intolerance of the guideline-recommended prednisone therapy. Subsequent complete metabolic panel showed stable renal function, albumin improvement to 4.1 g/dL, and total protein improvement to 6.4 g/dL. Notes from the PCP stated that the patient was still experiencing significant lower extremity swelling attributed to her underlying chronic lower extremity edema rather than to her MCD. Notable additional complications from the patient’s condition included new-onset hyperlipidemia and steroid-induced diabetes. The patient is receiving ongoing outpatient care with PCP, nephrology, and cardiology at the time of this report.

## Discussion

This case demonstrates a coexistence of nephrotic syndrome and chronic lower extremity edema delaying outpatient workup for nephrotic syndrome. Lower extremity edema is extremely prevalent in the United States, with as high as 19.1% of US adults having this condition [5]. It can also be age-related, with individuals over 80 years old experiencing 2.9-5 times the odds of reporting lower extremity edema [5]. Edema is also associated with a sedentary lifestyle, low income, obesity, and many other conditions [5]. This could easily lead a clinician to dismiss the significance of lower extremity edema and attribute it to a functional status rather than an underlying condition. This may be especially true when the differential for what causes lower extremity edema is extremely broad and can be multifactorial. Causes of bilateral lower extremity edema can include venous insufficiency, heart failure, lymphedema, hypoalbuminemia, thyroid disorders, drugs, and much more [6]. Hypoalbuminemia is a common laboratory result in the inpatient setting. Serum albumin decreases with increased vascular permeability in acute inflammation [7]. It is not uncommon to see patients in both the inpatient and outpatient settings with hypoalbuminemia and lower extremity edema that is not attributed to nephrotic syndrome. Low albumin and edema alone may not lead a clinician to suspect nephrotic syndrome, especially in older populations. Identifying the cause of edema requires a thorough history and physical examination and is crucial to identifying and treating the underlying condition appropriately. Further, clear documentation of the characteristics and extent of chronic edema over time is needed to identify changes, including progression, as was the case in our patient, that may suggest additional or alternative diagnoses.

Nephrotic syndrome is an especially rare cause of edema in adults, with a reported incidence of 3 new cases per 100,000 [8]. As discussed earlier, MCD is an even smaller subset, accounting for only 10-15% of nephrotic syndrome [3], making this case particularly unique. In addition, the median age of adult-onset MCD was reported to be 43 years [9], whereas our patient presented with new-onset MCD at age 79. The rarity of this presentation further underscores how easily the correct diagnosis could be missed. However, correct and timely diagnosis and treatment are important in preserving kidney function. Older adult-onset MCD seems less likely to relapse than younger-onset MCD; however, the rate of remission in older adults is lower [9].

The mainstay of treatment is glucocorticoids, which leads to remission in 80-95% of adults [10]. Length of time to complete remission varies from four weeks to four months of therapy [10]. Relapses can occur, and patients can be resistant to glucocorticoids or develop complications from the glucocorticoid therapy. Additional or alternative steroid-sparing immunosuppressive therapy is available. One trial highlighted the efficacy of combined tacrolimus and low-dose steroid therapy, as was used in our patient, against high-dose steroid therapy alone, suggesting it may reduce the risk of relapse [11].

There should also be significant consideration of the comorbidities caused directly by nephrotic syndrome, as well as the unintended side effects of its treatment. In particular, our patient experienced new-onset hyperlipidemia from nephrotic syndrome and diabetes secondary to prednisone use. Our patient’s HbA1c increased from a baseline of 5.4% to 6.5% after initiation of prednisone therapy. Our patient’s baseline lipid panel showed total cholesterol of 165 mg/dL, triglycerides of 78 mg/dL, low-density lipoprotein (LDL) of 81 mg/dL, and high-density lipoprotein (HDL) of 68 mg/dL, with repeat lipid panel after symptom onset showing total cholesterol of 383 mg/dL, triglycerides of 195 mg/dL, LDL of 248 mg/dL, and HDL of 97 mg/dL. Impaired regulation of lipid metabolism is very common in nephrotic syndrome [12]. It is important to identify this risk factor in addressing the patient’s overall cardiovascular health, as patients with nephrotic syndrome are already at an increased risk of myocardial infarction [12]. Other notable risks associated with nephrotic syndrome are thromboembolism and acute kidney injury [12]. The side effects of glucocorticoid therapy are well known among clinicians and include adrenal insufficiency and insulin resistance, potentially leading to diabetes, the latter of which occurred in our patient. As most patients with MCD are treated for extended durations (as patients are not considered steroid-resistant until 16 weeks without response [10]), these are significant considerations. While following treatment response in the outpatient setting, it is important for the clinician to continue to monitor for these potential adverse effects to minimize the patient’s long-term cardiovascular risk.

## Conclusions

This case highlights the importance of keeping a broad differential for patients with lower extremity edema, regardless of their age profile. MCD can occur at any age, and the workup for progressive lower extremity edema should include evaluation of proteinuria and serum albumin levels. Nephrotic syndromes carry substantial secondary risks to patients, including cardiovascular and thromboembolic events, dyslipidemia, and kidney injury. Treatment is focused on glucocorticoids, but there are additional adverse effects from treatment that need to be monitored by the clinician. Recovery is a prolonged course that often includes relapse.
